# Multiple evolutionary processes drive the patterns of genetic differentiation in a forest tree species complex

**DOI:** 10.1002/ece3.421

**Published:** 2013-01-10

**Authors:** Rebecca C Jones, Dorothy A Steane, Martyn Lavery, René E Vaillancourt, Brad M Potts

**Affiliations:** 1 School of Plant Science, University of TasmaniaPrivate Bag 55, Hobart, Tasmania, 7001, Australia; 2 CRC for ForestryPrivate Bag 12, Hobart, Tasmania, 7001, Australia; 3 Faculty of Science, Health, Education and Engineering, University of the Sunshine CoastSippy Downs, Queensland, Australia; 4 Arianda Pty LtdPO Box 732, Williamstown, Victoria, 3016, Australia

**Keywords:** Blue gum, clinal variation, conservation genetics, evolution, gene pool management, genetic diversity, hybridization, microsatellite DNA, speciation

## Abstract

Forest trees frequently form species complexes, complicating taxonomic classification and gene pool management. This is certainly the case in *Eucalyptus*, and well exemplified by the *Eucalyptus globulus* complex. This ecologically and economically significant complex comprises four taxa (sspp. *bicostata*, *globulus*, *maidenii*, *pseudoglobulus*) that are geographically and morphologically distinct, but linked by extensive “intergrade” populations. To resolve their genetic affinities, nine microsatellites were used to genotype 1200 trees from throughout the natural range of the complex in Australia, representing 33 morphological core and intergrade populations. There was significant spatial genetic structure (*F*_ST_ = 0.10), but variation was continuous. High genetic diversity in southern ssp. *maidenii* indicates that this region is the center of origin. Genetic diversity decreases and population differentiation increases with distance from this area, suggesting that drift is a major evolutionary process. Many of the intergrade populations, along with other populations morphologically classified as ssp. *pseudoglobulus* or ssp. *globulus*, belong to a “cryptic genetic entity” that is genetically and geographically intermediate between core ssp. *bicostata*, ssp. *maidenii,* and ssp. *globulus*. Geography, rather than morphology, therefore, is the best predictor of overall genetic affinities within the complex and should be used to classify germplasm into management units for conservation and breeding purposes.

## Introduction

The most widely accepted species concept defines species as reproductively isolated units (Coyne and Orr 2004). However, for many organisms, morphological and ecological differences among recognized species are not always accompanied by reproductive isolation (Stebbins 1989) and morphological boundaries between species can be blurred by hybridization and introgression, resulting in species complexes that are taxonomically challenging. This is seen in diverse plant groups ranging from orchids and sunflowers (Dressler and Dodson 1960; Rieseberg et al. 1991) to forest trees such as the oaks and eucalypts (Johnson 1976; Manos and Fairbrothers 1987; Griffin et al. 1988). The use of morphological traits to define taxa in species complexes can also be problematic, as homology (similarity due to common ancestry) and homoplasy (similarity due to convergence) cannot be distinguished. Hence, in the last few decades, molecular markers have been used to assess genetic differentiation and evolutionary histories of species complexes.

Molecular studies have often identified low levels of genetic divergence among taxa in species complexes, implying recent divergence and/or hybridization and introgression. Particularly well-studied species complexes include the sunflowers (e.g., Beckstrom-Sternberg et al. 1991; Rieseberg et al. 1991; Yatabe et al. 2007) and oaks (e.g., Hokanson et al. 1993; Hess and Stoynoff 1998; Gomory et al. 2001; Kashani and Dodd 2002; Aldrich et al. 2003). Molecular markers have been used to elucidate phylogenetic relationships among closely related taxa in other forest tree species complexes such as *Abies magnifica*–*Abies procera* (Oline 2008), *Melaleuca quinquenervia* (Cook et al. 2008), *Astronium* (Caetano et al. 2008), *Fagus sylvatica* (Gomory et al. 2007), *Acacia* (Byrne et al. 2002; Millar et al. 2011), and *Eucalyptus* (reviewed by Grattapaglia et al. 2012). Recent advances in Bayesian assignment procedures can prove useful in defining taxonomic units in species complexes (e.g., Gomory et al. 2007; Caetano et al. 2008; Duminil and Di Michele 2009; Millar et al. 2011). Duminil et al. (2006) even argued that the best approach when studying closely related taxa is to use only Bayesian procedures for species delimitation, without reference to morphological data.

**Figure 1:**
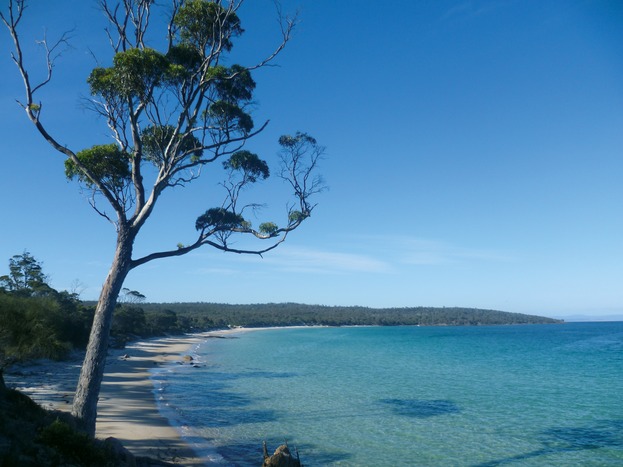
*Eucalyptus globulus* ssp. *globulus* at Coles Bay, on the east coast of Tasmania.

The speciose Australian tree genus *Eucalyptus* includes many species complexes, where poor morphological resolution of recognized taxa is thought to be due, at least in part, to recent and ongoing speciation in some lineages (Byrne 2008), coupled with contemporary and historic hybridization (Griffin et al. 1988; McKinnon et al. 2001). We here report a molecular study of the *Eucalyptus globulus* species complex (Fig. 1) aimed at (1) understanding the pattern of genetic variation and the evolutionary processes that have shaped genetic differentiation and (2) providing a framework for the management of this important genetic resource. This complex is of ecological significance as it dominates many lowland forests of southeastern Australia. It is a foundation species in these forests and many insects and birds, including the endangered swift parrot (*Lathamus discolor*), feed on the nectar of its flowers (Hingston et al. 2004). The species complex is also of economic importance to the global pulp and paper industry because it includes the world's most widely planted temperate hardwood species (Eldridge et al. 1993).

Conserving the maximum amount of genetic diversity in a species, thereby maximizing its long-term adaptive flexibility (Frankel and Soule 1981), has been a priority of conservation strategies, and recent papers have called for evolutionary principles to be incorporated into conservation efforts (Sgrò et al. 2010), particularly in the era of climate change (Mergeay and Santamaria 2012). In the case of foundation species such as *E. globulus*, these strategies will also help to conserve associated biodiversity (Barbour et al. 2009; Whitham et al. 2010). However, a broad-based molecular genetics framework is also required to guide the management of this native genetic resource, particularly with respect to the risk of genetic contamination from plantation establishment (Potts et al. 2003; Barbour et al. 2008). The risk of contamination of native forest gene pools from interspecific hybridization with locally exotic plantation species has been well studied (Laikre et al. 2010). F_1_ hybrids have been detected in open-pollinated seed collected from native species adjacent to *E. globulus* plantations and, while rare at this stage, F_1_ hybrids have also been found established in the wild (Barbour et al. 2008). Interspecific hybridization is often readily detected through simple morphological observations (Barbour et al. 2005). However, assessing the risk of gene flow from *E. globulus* plantations established within the native range of the species complex is more difficult and, as a first step, requires an understanding of the differentiation between the germplasm used in plantations and the adjacent native forest.

**Figure 2:**
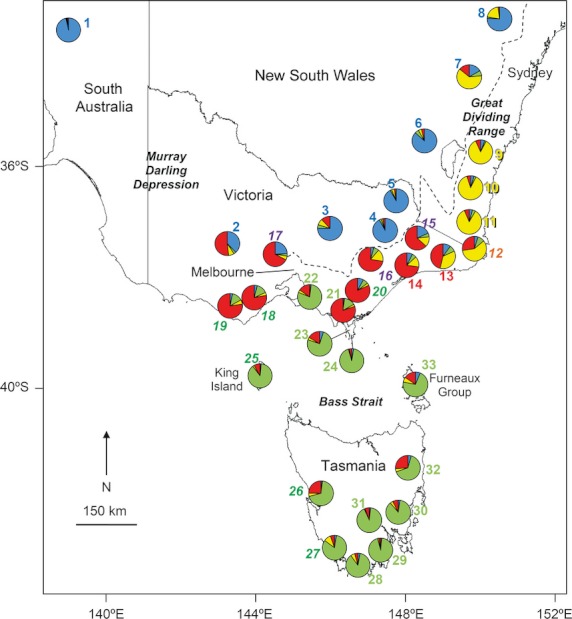
Regions of the *Eucalyptus globulus* complex used in this study. Regions are numbered (for further details see Table 1) and the color of the number corresponds to the morphological affinities of the region: blue = *bicostata*, yellow = *maidenii*, orange = *maidenii*–*pseudoglobulus* intergrade, red = *pseudoglobulus*, purple = *bicostata–pseudoglobulus* intergrade, dark green = *globulus* intergrade, light green = *globulus*. Intergrade region numbers are also italicized. The three major geographic barriers are shown in bold italics: the Murray Darling Depression (lowland plains with a warm, semi-arid climate), the Great Dividing Range (indicated by a dashed line), and the Bass Strait (channel that separates the island of Tasmania from the rest of Australia). Pie charts show the average proportion of membership of each of the 33 regions into each of the clusters (*K* = 4), from the STRUCTURE analysis of all sampled individuals of the *E. globulus* complex.

The *E. globulus* complex comprises four taxa of forest trees that form a monophyletic group (McKinnon et al. 2008; Steane et al. 2011) and are variously treated as species or subspecies (*bicostata*, *globulus*, *maidenii,* and *pseudoglobulus*; Pryor and Johnson 1971; Kirkpatrick 1975; Jordan et al. 1993; Brooker 2000), but most recently as subspecies (Slee et al. 2006). These taxa will hereafter be referred to by their subspecific names, hence *globulus* refers to the subspecies and *E. globulus* refers to the species complex. The *E. globulus* complex has a largely continuous distribution in southeastern Australia, although there are several small, disjunct populations at the periphery of the species' range (Brooker and Kleinig 2006; Fig. 2). Three major geographic barriers provide significant contemporary barriers to dispersal: the Bass Strait, the Great Dividing Range, and the Murray Darling Depression (Fig. 2). However, while the core distributions of the four *E. globulus* taxa are geographically separated, the taxa – as defined taxonomically – span these major contemporary barriers. Differences in flowering time are also expected to provide barriers to gene flow among populations (Jones et al. 2011).

Morphologically, the four *E. globulus* taxa are differentiated mainly by capsule traits: *maidenii* has the smallest fruit in the complex and up to seven fruit per umbel; *bicostata* and *pseudoglobulus* are both three-fruited, with *pseudoglobulus* having smaller fruit, longer pedicels, and fewer ribs on the capsules than *bicostata*; *globulus* has large, solitary capsules. The geographic cores of these four taxa are morphologically distinct, but are linked by “intergrade” populations that are intermediate in morphology (Kirkpatrick 1975). These intergrade populations complete a continuum in capsule morphology across the geographic range of *E. globulus*. The origins of the intergrade populations, as well as the origin of the species complex and migration pattern, have been the subjects of considerable debate (Kirkpatrick 1975; Jordan et al. 1993; Wallis et al. 2011). The intergrade populations occur across a wide area (Fig. 2), representing a significant proportion of the distribution of the species complex, and are therefore of ecological as well as evolutionary importance. Furthermore, their diverse and intermediate morphologies confound taxonomic classification and cause difficulties in managing these forests and genetic resources. The intergrade populations may originate from: (1) primary differentiation (divergence within a continuous series of populations along a selective gradient), or (2) secondary intergradation (hybridization and introgression between previously isolated gene pools), although the possibility of a more complex scenario involving both processes cannot be dismissed (Jordan et al. 1993; Wallis et al. 2011). These two evolutionary scenarios are difficult to distinguish based on current patterns of variation (Endler 1977). However, a zone of recent secondary intergradation would be expected to display increased diversity due to gene flow from differentiated populations (Rieseberg and Wendel 1993). For example, molecular data show that the genetic diversity within a *Populus fremontii*–*Populus angustifolia* hybrid zone is much higher than in the pure species zones (Whitham et al. 1999). In addition, a recent hybrid zone would be expected to have correlated morphological and molecular affinities, as was the case in the *Quercus crassifolia*–*Quercus crassipes* hybrid zone in Mexico (Tovar-Sanchez and Oyama 2004), because linkage disequilibrium is expected to decay slowly in long-lived organisms.

Genetic variation across parts of the *E. globulus* species complex has been investigated in several taxonomic, quantitative, and molecular genetic studies (reviewed by Potts et al. 2004; see also McKinnon et al. 2005; Steane et al. 2006; Hamilton et al. 2011; Kulheim et al. 2011; Stackpole et al. 2011; Wallis et al. 2011; Yeoh et al. 2012; Dutkowski and Potts 2012). However, most of these studies have focused on *globulus* and its intergrade populations, as these have been of most interest to breeding programs and the plantation industry. Very few, or no, samples of *pseudoglobulus*, *bicostata,* or *maidenii* or their intergrade populations were included in these genetic studies. In addition, selection acting on morphological and other quantitative traits (e.g., Steane et al. 2006) could cause a misrepresentation of true genetic relationships in the species complex. This study therefore uses putatively neutral nuclear DNA markers (microsatellites) to understand the pattern of genetic variation and the evolutionary processes that have shaped genetic differentiation in the *E. globulus* gene pool. To achieve this, we genotyped one of the largest samples yet of a forest tree species and coupled these results with morphological measurements to determine the evolutionary origin of intergrade populations.

## Materials and Methods

### Plant material

Leaf tissue for DNA extraction, a herbarium specimen (including buds and capsules), and open-pollinated seed (where available) were sampled from 1200 adult trees representing 103 natural populations, 33 regions, and the four taxa of the *E. globulus* species complex, including their intergrades (Fig. 2, Table S1). Trees sampled were at least 100 m apart, to avoid sampling closely related trees (see Skabo et al. 1998; Jones et al. 2007). Localities of *globulus* and its intergrades were grouped into races (as defined by Dutkowski and Potts 1999), hereafter referred to as “regions”; the other taxa and their intergrades were grouped into geographic regions based on overall morphology (Table S1). Populations were classified into a pure taxon or intergrade type following the Brooker and Kleinig (2006) field guide and/or the collections of Kirkpatrick (1975) and Jordan et al. (1993) (Table S1).

### Molecular methods

#### DNA extraction and PCR

Total genomic DNA was extracted from fresh or frozen leaf tissue using a CTAB method (Doyle and Doyle 1990) with several modifications (McKinnon et al. 2004b). DNA quality and quantity were assessed by gel electrophoresis and comparison with a Lambda *Hin*dIII molecular weight standard.

Nine microsatellite loci were selected; six (EMCRC) designed by Steane et al. (2001), two (EMBRA11 and EMBRA19) by Brondani et al. (1998), and one (EMBRA30) by Brondani et al. (2002), and the primer sequences are available in each of these references. These loci have been mapped and there is no evidence for linkage between the loci (J. S. Freeman, pers. comm.). Forward primers were synthesized to include a WellRED fluorescent label (Sigma-Aldrich Pty Ltd, Castle Hill, NSW, Australia) that enables detection on a CEQ™ fragment analysis system (Beckman Coulter Inc., Fullerton, California). PCR conditions followed Brondani et al. (1998) for the three EMBRA loci, and Jones et al. (2002) for the EMCRC loci except that 0.28 μmol/L of each primer was used in a total reaction volume of 12.5 μL. PCRs were performed separately and PCR products were co-loaded in two sets (Set 1: EMCRC5, EMCRC6, EMCRC7, EMBRA19; Set 2: EMCRC2, EMCRC10, EMCRC11, EMBRA11, EMBRA30) and sized by comparison with the CEQ™ DNA Size Standard-400 using the Beckman Coulter Inc. Fragment Analysis software for allele binning. To allow data sets from Jones et al. (2002), Steane et al. (2006), Jones et al. (2007), and Foster et al. (2007) to be combined with results from this study, control samples were included (see Data S1; repeatability and missing data percentages are also given in Data S1). Only samples that yielded results for six or more loci were retained, resulting in a final data set of 1200 individuals representing 103 localities, 33 regions, and all four taxa of the *E. globulus* species complex, including their intergrades, across the entire natural range of the species complex (Fig. 2, Table S1).

#### Molecular data analysis

All statistics calculated at the locality or regional level were based on the 103 localities or the 33 regions shown in Fig. 2 and listed in Table S1.

A variety of genetic diversity statistics were used to assess and compare genetic variation within populations. GDA 1.1 (Lewis and Zaykin 2001) was used to calculate the following genetic diversity parameters, averaged for each locus and region: number of alleles (*A*), observed and expected heterozygosity (*H*_O_ and *H*_e_, respectively), and Wright's Fixation Index (*F*). Null allele frequencies in each region were estimated using the Population Inbreeding method in INEst (Chybicki and Burczyk 2009). Allelic richness (El Mousadik and Petit 1996) was calculated at the region and locality levels, using FSTAT (Goudet 2002). Contours of allelic richness per locality were plotted on a map using 3DField 2.9.6 (Galouchko 2007).

To determine the relationships among regions, F-statistics (FIS, FIT, FST, Weir and Cockerham 1984) and Nei's (1972) genetic distance were calculated. A pairwise matrix of Nei's (1972) genetic distance among regions, calculated using GDA 1.1, was used to construct a Neighbor-Joining (Saitou and Nei 1987) radial tree with proportional branch lengths, using T-REX 4.0 (Makarenkov 2001). *F*-statistics were calculated for each locus at the regional level, using GDA 1.1. *F*_ST_ was also calculated for each taxon at the regional level.

To determine the relationships among the four taxa, pairwise *F*_ST_ values among taxa were calculated using FSTAT (Goudet 2002), based on the “core” localities indicated in Table S1. The cores were based on morphology, geography, and prior analyses with STRUCTURE (i.e., individuals from geographically disjunct localities, morphological intergrade localities, and intermediate localities from STRUCTURE analyses were excluded from the analysis).

The number of groups of genetically similar individuals (*K*) in the *E. globulus* complex, and the affinities of individuals to these groups (*Q*) independent of *a priori* morphological assumptions, were determined using STRUCTURE 2.2.3 (Pritchard et al. 2000) and the Δ*K* method described by Evanno et al. (2005). Assuming no prior population groupings and using the admixture model, the estimated *K* was determined by comparing the estimated log probability of data at different values of *K* (from *K* = 1 to *K* = 20), using 100 000 MCMC repetitions following a burnin of 120 000 repetitions (at which point stationarity had been reached). Twenty independent runs for each value of *K* were performed, and results were interpreted using the five runs with the highest likelihoods. The *Greedy* algorithm in CLUMPP (Jakobsson and Rosenberg #b^501^) was used to derive a single output from the five independent runs at each *K* from *K* = 2 to *K =* 6. DISTRUCT (Rosenberg 2004) was used to display the probability of membership (*Q*) of each individual into each of the inferred clusters. The average proportion of membership of each of the 33 regions into the *K* = 6 clusters, from the analysis of all sampled individuals of the *E. globulus* complex, was plotted on a map.

A second STRUCTURE analysis was undertaken to directly compare molecular and morphological affinities of individuals. This analysis used a model with *a priori* population information (i.e., POPFLAG = 1) for populations of “core” *globulus*, *bicostata*, *pseudoglobulus,* and *maidenii* (based on morphology and geography, see Table S1). An admixture model was used to assign individuals from intergrade and geographically outlying populations into the clusters defined as core *globulus*, *bicostata*, *pseudoglobulus,* and *maidenii*, and the original “core” individuals were also reassigned to the four clusters using an admixture model (i.e., POPFLAG = 0). These core individuals were therefore duplicated in the analysis. Ten independent runs at *K* = 4 were performed using 50 000 MCMC repetitions and a burnin of 50 000 repetitions, and the individual *Q*-values from the run with the highest likelihood were used to test for correlations with morphological affinities (see below) and were not presented graphically. Only individuals for whom both morphological and molecular data were available were included in this analysis.

### Morphological methods

**Figure 3:**
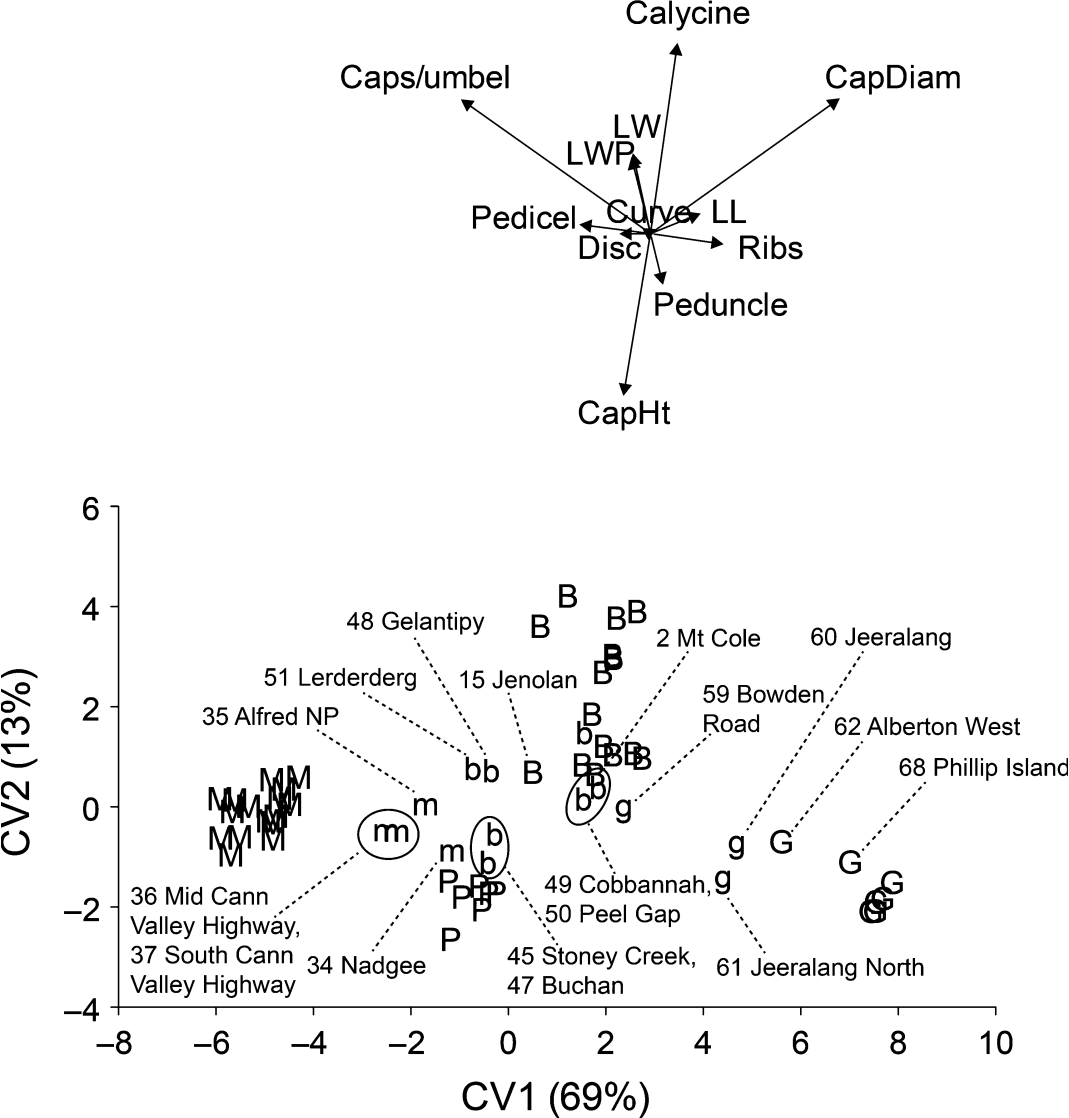
Plots of *Eucalyptus globulus* locality mean canonical variate scores in the space defined by the region level analysis, using 12 capsule, inflorescence and leaf variables. CapDiam, capsule diameter; CapHt, total capsule height; Disc, disc height; Calycine, calycine ring height; Ribs, number of ribs per capsule; Pedicel, pedicel length; Peduncle, peduncle length; Caps/umbel, mean number of capsules per umbel; LW, lamina width; LWP, length to widest point of leaf; LL, lamina length; Curve, leaf curvature (length of a line drawn from the primary vein perpendicular to a line connecting the lamina base to the lamina tip).

For 446 of the trees sampled in this study, 12 capsule, inflorescence, and leaf variables (defined in Fig. 3) were measured following the descriptions of Kirkpatrick (1975), Jordan et al. (1993), and Jones et al. (2002). Five average-sized umbels with all parts intact were selected from the youngest age class (1 year old) of mature capsules, and the middle capsule of each umbel (where applicable) was measured. Inflorescence data were collected from up to 70 umbels per tree. Where abortion scars were present on the umbel, or capsules had obviously dislodged during sampling, capsules were scored as present. Leaf measurements were taken on a single representative leaf. Measurements were combined with those from Jones et al. (2002).

Analyses were undertaken using the means of the five capsule replicates from each tree. The variables were transformed to optimize population normality and remove the correlation between locality or region means and variances. The transformations were the same as in Jordan et al. (1993), with four exceptions: length to widest point of leaf (LWP) (log), Pedicel (square root), Ribs (square root), and Curve (*x*^0.75^). Multivariate analyses were undertaken at the region level using Canonical Discriminant Analysis, an ordination technique that maximizes differences between groups relative to within-group variation, using the PROC DISCRIM procedure in the statistical package SAS (Version 9.1; SAS Institute, Cary, North Carolina). The locality level mean canonical variate scores were placed within the multivariate discriminant space defined by the region level analysis. The vectors indicating the direction of variation in each variable were constructed from the standardized canonical coefficients. The lengths of the vectors were proportional to the analysis of variance (ANOVA) *F*-values (see Jordan et al. 1993), calculated between groups for each variable. Pairwise Mahalanobis (morphological) distances among regions and significance (*P* < 0.05) were calculated using PROC DISCRIM.

### Relationship between morphological, geographic, and molecular affinities

To test for the effects of drift and selection, a Mantel test was conducted using XLSTAT (Version 2006.5; Addinsoft SARL, Paris, France) to test the relationships between Mahalanobis distance (calculated from morphological data), Nei's (1972) genetic distance (calculated from microsatellite data), and geographic distance (calculated from latitude/longitudes in GenAlEx 6.2, Peakall and Smouse 2006) among regions.

Using morphological data, the core *bicostata*, *globulus*, *pseudoglobulus,* and *maidenii* individuals (see Table S1) were used to define the multivariate space, and the intergrade/geographically outlying individuals were fitted within this space. The morphological affinities (*M*) of these individuals to each of the core taxa (B, G, P, M) were then calculated using PROC DISCRIM (i.e., *M*_B_, *M*_G_, *M*_P_, *M*_M_) based on the classification functions derived from PROC DISCRIM. PROC CORR was then used to test whether the morphological affinities of individuals were significantly correlated with their molecular affinities, in each region in Victoria and NSW with more than 10 individuals, using Pearson's correlation coefficient. The molecular affinities had been calculated in the STRUCTURE analysis (above) that used *a priori* population information for the same core individuals, and calculated the affinities of individuals (*Q*) to each of the four core groups (i.e., *Q*_B_, *Q*_G_, *Q*_P_, *Q*_M_).

## Results

### Genetic diversity

**Figure 4:**
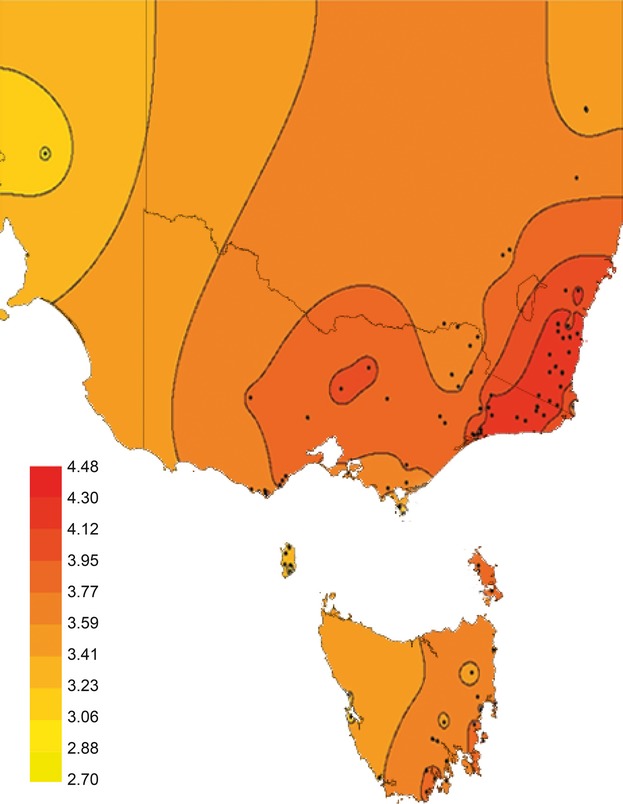
Allelic richness (*R*_t_) for localities of *Eucalyptus globulus*, plotted as a contour map.

General diversity statistics for the microsatellite loci are given in Table S2 and Data S2. At the population level, genetic diversity, as measured by either expected heterozygosity (*H*_e_) or allelic richness (*R*_t_), was highest in the 11-South East Forests region, in the southern part of the *maidenii* distribution (Table 1). There was a weak but significant negative linear correlation between geographic distance from this region (29-Yurammie, a locality within the 11-South East Forests region, 36.9°S, 149.72°E) and genetic diversity, at both the region and locality level (*H*_e_ 33 regions *r*^2^ = 0.31, *P* = 0.0008; *R*_t_ 33 regions *r*^2^ = 0.34, *P* = 0.0004; *H*_e_ 93 localities *r*^2^ = 0.34, *P* < 0.0001; *R*_t_ 93 localities *r*^2^ = 0.37, *P* < 0.0001). This was driven partly by the large geographic disjunction and low genetic diversity of the Mt. Bryan locality as well as the lower diversity in Tasmania, but when the Mt. Bryan population and Tasmanian samples were excluded, the correlations were still significant at both the regional and locality levels (data not shown). As the distribution of *E. globulus* is interrupted by geographic barriers (see Fig. 2) and migration is likely be non-linear, contour plotting of allelic richness was used to reveal the areas of high genetic diversity (Fig. 4). The primary center of diversity was in the 11-South East Forests region, with secondary centers on the east coast of Tasmania and on the western side of the Great Dividing Range. The pattern was the same for expected heterozygosity at the locality level, and for both allelic richness and expected heterozygosity at the regional level (data not shown). Genetic diversity in geographically disjunct regions of the species complex was lower than the mean for the species complex (e.g., 1-Mt. Bryan *H*_e_ = 0.57, 8-Nullo Mountain *H*_e_ = 0.68, 25-King Island *H*_e_ = 0.65; mean for *E. globulus H*_e_ = 0.78, Table 1), although some populations in the continuous range also exhibited low levels of genetic diversity (e.g., 24-Wilson's Promontory *H*_e_ = 0.65, 23-Tidal River *H*_e_ = 0.69, Table 1).

**Table 1:** Genetic diversity parameters for *Eucalyptus globulus*, calculated at the regional level

Region (taxon code)	Distribution	*n*	*A*	*H*_e_	*H*_o_	*R*_t_	*F*
1-Mt. Bryan (B)	Disjunct	24.0	4.6	0.57	0.48	4.11	0.17
2-Mt. Cole (B)	Disjunct	18.7	8.6	0.80	0.67	7.15	0.17
3-Hume (B)		29.0	10.8	0.84	0.72	8.13	0.14
4-Omeo (B)		21.9	8.9	0.77	0.66	6.92	0.15
5-Northeastern Victoria (B)		31.2	9.8	0.77	0.60	6.97	0.23
6-Canberra (B)		19.1	7.3	0.76	0.65	6.27	0.16
7-Jenolan (B)	Disjunct	20.1	7.1	0.77	0.65	6.21	0.15
8-Nullo Mountain (B)	Disjunct	19.8	6.8	0.68	0.59	5.63	0.14
*bicostata* mean		23.0	8.0	0.75	0.63	6.42	0.16
9-Araluen (M)		38.6	14.6	0.85	0.73	9.14	0.14
10-Wadbilliga (M)		112.9	20.2	0.85	0.73	9.44	0.15
11-South East Forests (M)		69.6	17.4	0.87	0.74	9.54	0.15
*maidenii* mean		73.7	17.4	0.86	0.73	9.37	0.15
12-Alfred-Nadgee (m)		43.8	15.0	0.86	0.68	9.12	0.21
m intergrade mean		43.8	15.0	0.86	0.68	9.12	0.21
13-East Gippsland (P)		25.0	12.7	0.86	0.71	9.40	0.18
14-Lakes Entrance (P)		46.9	13.7	0.87	0.73	8.82	0.16
*pseudoglobulus* mean		35.9	13.2	0.86	0.72	9.11	0.17
15-Buchan (b)		35.7	13.7	0.86	0.69	9.14	0.21
16-Mitchell River (b)		18.8	9.7	0.81	0.71	7.84	0.12
17-Lerderderg (b)		19.8	9.3	0.82	0.68	7.59	0.17
b intergrade mean		24.7	10.9	0.83	0.69	8.19	0.17
18-Eastern Otways (g Vic)		27.3	10.6	0.79	0.70	7.99	0.12
19-Western Otways (g Vic)		30.4	11.2	0.79	0.70	7.87	0.11
20-Strzelecki Ranges (g Vic)		22.0	9.0	0.79	0.69	7.29	0.13
g intergrade (Vic) mean		26.6	10.3	0.79	0.70	7.72	0.12
21-South Gippsland (G Vic)		27.8	9.9	0.77	0.68	7.15	0.12
22-Phillip Island (G Vic)		23.3	10.2	0.77	0.71	7.51	0.08
23-Tidal River (G Vic)		29.7	7.3	0.69	0.76	5.62	−0.10
24-Wilson's Promontory Lighthouse (G Vic)		28.6	5.8	0.65	0.64	4.67	0.00
*globulus* (Vic) mean		27.3	8.3	0.72	0.70	6.24	0.03
25-King Island (g Tas)	Disjunct	36.9	7.9	0.65	0.56	5.76	0.14
26-Western Tasmania (g Tas)	Disjunct	31.0	9.1	0.75	0.63	6.69	0.16
27-Port Davey (g Tas)	Disjunct	33.8	9.8	0.74	0.64	6.64	0.13
g intergrade (Tas) mean		33.9	8.9	0.71	0.61	6.36	0.14
28-Recherche Bay (G Tas)		25.4	9.2	0.79	0.68	7.19	0.15
29-Southern Tasmania (G Tas)		23.9	8.9	0.81	0.74	7.08	0.10
30-Southeastern Tasmania (G Tas)		67.7	14.6	0.83	0.69	8.23	0.16
31-Dromedary (G Tas)		17.0	8.1	0.78	0.72	6.96	0.09
32-Northeastern Tasmania (G Tas)		88.6	14.4	0.83	0.69	8.24	0.17
33-Furneaux (G Tas)	Disjunct	43.9	11.1	0.80	0.69	7.49	0.15
*globulus* (Tas) mean		44.4	11.1	0.81	0.70	7.53	0.14
*E. globulus* mean		34.9	10.5	0.78	0.68	7.3	0.14

Disjunct regions (isolated from other populations by more than 80 km, and those on the Bass Strait Islands) are indicated; all other regions are within the continuous range of the *E. globulus* distribution.

*n* = average number of samples per region, *A* = observed number of alleles per locus, *H*_e_ = expected heterozygosity, *H*_o_ = observed heterozygosity, *R*_t_ = allelic richness, *F* = Wright's Fixation Index.

Recent hybrid origin would be expected to impart intergrade populations with increased genetic diversity. Genetic diversity in these intergrade populations was not higher than in their putative parental populations. For example, 15-Buchan, 16-Mitchell River, and 17-Lerderderg, which were intermediate in morphology between *pseudoglobulus* and *bicostata*, did not have higher diversity (*H*_e_ = 0.86, 0.81 and 0.82, respectively, Table 1) than the pure populations of *pseudoglobulus* (mean *H*_e_ = 0.86, Table 1) and *bicostata* (*H*_e_ = 0.75, Table 1). Similarly, but at a finer scale, the mid Cann Valley Highway locality (locality 36) had lower expected heterozygosity than the localities at the northern (locality 31, *maidenii*) and southern (locality 37, *maidenii*–*pseudoglobulus* intergrade) ends of the highway (data not shown).

#### Population differentiation

Most of the genetic variation in the complex was between individuals within regions; however, there was a significant proportion of genetic variation maintained between the regions. Considering region as the subpopulation, overall *F*_ST_ (i.e., inbreeding within a region relative to the entire species complex) was 0.10 (Table S2).

Differentiation was lowest among regions of *maidenii* (*F*_ST_ = 0.011, *n* = 3) and *pseudoglobulus* (*F*_ST_ = 0.024, *n* = 2) and highest among regions of *bicostata* (*F*_ST_ = 0.131, *n* = 8). Differentiation was moderate among regions of *globulus* (*F*_ST_ = 0.071, *n* = 10), increasing slightly when *globulus* intergrade regions were included (*F*_ST_ = 0.081, *n* = 16). Pairwise *F*_ST_ values among the four taxa revealed that *pseudoglobulus* was the least differentiated taxon (pairwise *F*_ST_ with other taxa 0.056–0.062, pairwise *F*_ST_ values among all taxa 0.056–0.112, data not shown).

**Figure 5:**
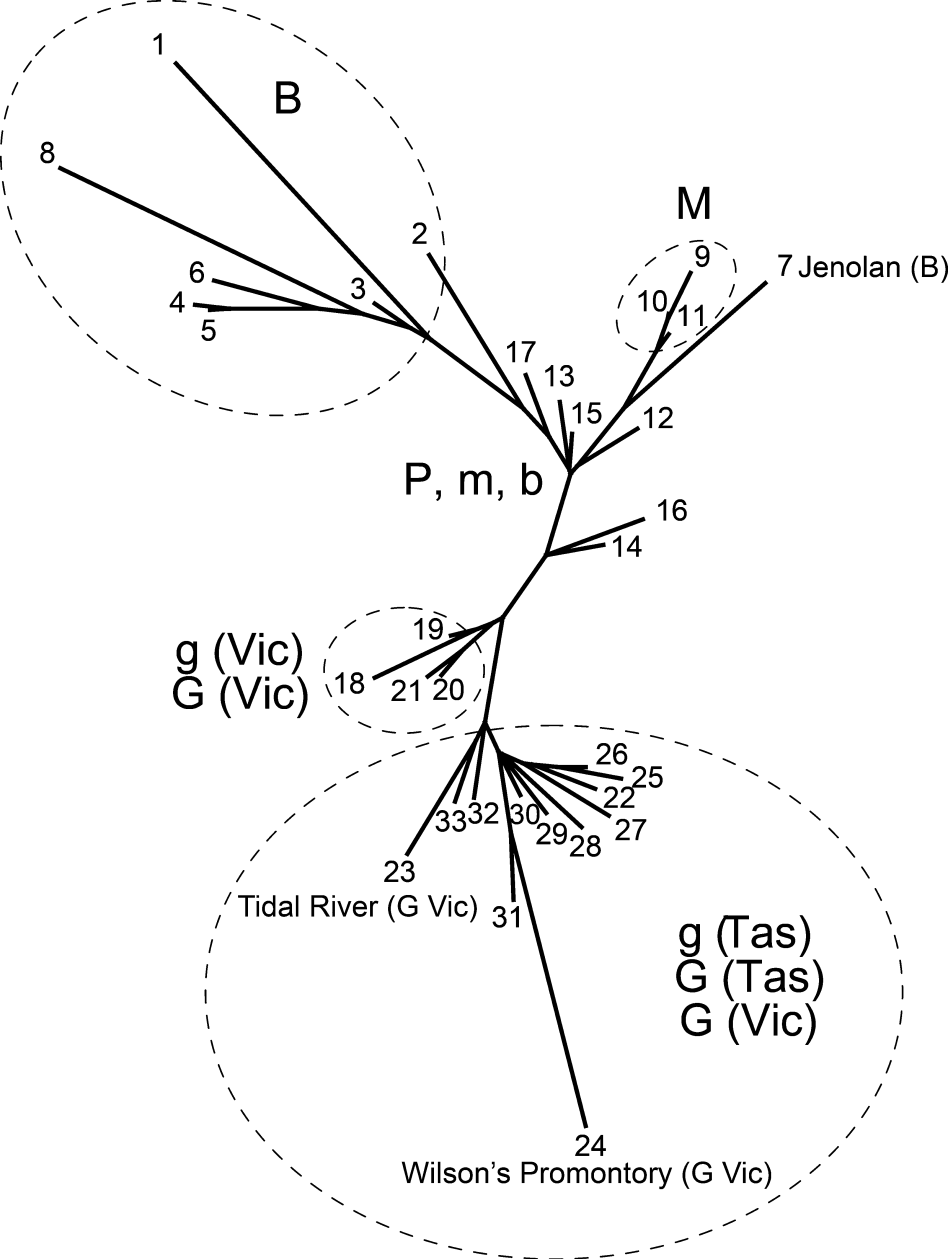
Neighbor-Joining radial tree with proportional branch lengths, based on a pairwise matrix of Nei's (1972) genetic distance between regions of the *Eucalyptus globulus* species complex. For definitions of region and taxon codes, see Table 1.

Regions of *globulus* and its intergrades clustered tightly together in the Neighbor-Joining radial tree, distinct from the *maidenii* cluster and the *bicostata* cluster (Fig. 5). Populations from the 11-South East Forests region, including *pseudoglobulus*, *maidenii,* and their intergrades, were in the center of the tree (Regions 9–15, Fig. 5). Disjunct populations of *bicostata* (1-Mt. Bryan and 8-Nullo Mountain) were on long branches, as was the population at 24-Wilson's Promontory (Fig. 5). 7-Jenolan, a region classified as *bicostata*, was basal to the *maidenii* branch (Fig. 5).

#### Individual affinities

**Figure 6:**
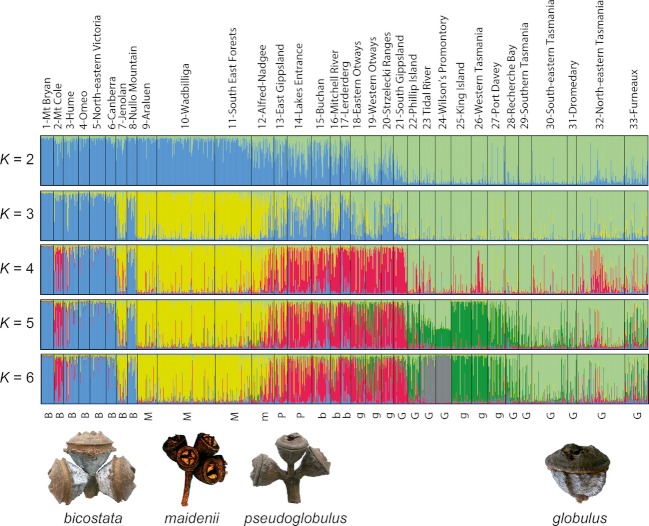
Proportion of membership for 1200 individuals of the *Eucalyptus globulus* species complex. Each individual is represented by a thin vertical line, partitioned into *K* colored segments representing the individual's estimated membership into the *K* genetic clusters. Individuals are grouped by region/race (indicated above the figure), and the overall capsule morphology of core regions is shown below the figure.

The Δ*K* method of Evanno et al. (2005) detected the major split in the entire *E. globulus* gene pool at *K* = 2 (Fig. S1), corresponding to a split of Tasmanian and mainland individuals, but with populations consisting of admixed individuals in inland central Victoria linking these two groups (Fig. 6). There was a secondary peak at *K* = 4 (Fig. S1), where three of the four morphological taxa were relatively well defined (Fig. 2). The fourth taxon, *pseudoglobulus*, emerged only at *K* = 7, but this solution was unstable, occurring in only three of the five highest likelihood runs (data not shown). At each increase in *K,* the group of regions that split into a new cluster were geographically contiguous, but at each given *K,* there were intermediate individuals that linked the *K* clusters (Fig. 6). At higher *K* values, solutions were unstable across runs, or the individuals split across the *K* clusters with few individuals strongly assigned to a single cluster, with no meaningful geographic pattern for the individuals that were strongly assigned (data not shown).

### Morphological relationships

Capsule diameter, calycine ring height, number of capsules per umbel, and capsule height were the most important variables in differentiating the localities of the *E. globulus* complex (Fig. 3). Although the taxa of the *E. globulus* complex were differentiated, they were linked by a morphological continuum of intergrade populations. The localities that had been collected as core *pseudoglobulus* clustered together, but this group was not highly differentiated from the rest of the complex, unlike the core localities of *maidenii*, *bicostata,* and *globulus* that formed the extremities of the variation in the ordination (Fig. 3). Rather, the *pseudoglobulus* localities had morphological affinities to the intergrade localities that form part of the continuum between *bicostata* and *maidenii* (Fig. 3).

### Relationship between geographic, morphological, and molecular affinities

There was a highly significant association between Nei's (1972) genetic distance and geographic distance (*r*^2^ = 0.370, *P* < 0.0001) indicating that isolation-by-distance and genetic drift is an important process in the *E. globulus* species complex. The major outliers on this plot (not shown) were the pairwise comparisons of regions that were separated by a strong geographic barrier over a short geographic distance (e.g., Great Dividing Range). There was no association between Mahalanobis distance (calculated from the morphological data) and geographic distance (*r*^2^ = 0.007, *P* > 0.05). Nei's (1972) genetic distance and Mahalanobis distance were weakly but significantly correlated (*r*^2^ = 0.042, *P* = 0.007). Outliers on this plot (not shown) were mainly pure Victorian *globulus* populations in South Gippsland compared with geographically proximal, but morphologically differentiated, intergrade populations, indicating that selection is acting over a steep gradient in this area.

As a recent hybrid zone would be expected to have correlated morphological and molecular affinities, the morphological and molecular affinities of individuals in regions of NSW and Victoria were tested for significant correlations in each region in which there was clear variation in morphological affinities. The only region that had a significant correlation between morphological and molecular affinities was 20-Strzelecki Ranges. In this region, there was a strong correlation between morphological and molecular affinity to Victorian *globulus* (*r* = 0.64, *P* = 0.0007, *n* = 24). Fourteen individuals had strong and similar morphological and molecular affinities to Victorian *globulus* in this region. No significant correlations involving the other taxa were observed and individuals with morphological and molecular affinities to Victorian *globulus* were not geographically clustered (data not shown).

## Discussion

This combined analysis of molecular and morphological data, sampled over a large scale and focusing on morphologically intermediate populations as well as “pure” populations, revealed that multiple evolutionary processes have driven the patterns of genetic differentiation in the *E. globulus* species complex. Across the broad geographic range, continuous molecular and morphological variation link the four recognized taxa. These continua appear to be driven by the interaction of the key evolutionary processes of genetic drift, selection, and, to a lesser extent, hybridization. These processes, occurring at multiple geographic locations and scales, result in a poor relationship between molecular and morphological affinities in many intergrade regions, which has implications for gene pool management and exploitation.

### Genetic drift

The neutral molecular data revealed that drift is a strong process at both broad and fine geographic scales in *E. globulus*. Across the broad geographic range, there is a continuum in molecular genetic variation and a significant and positive association between genetic and geographic distance. While weak, the observed relationship with distance (*r*^2^ = 0.37) is comparable to that reported in other eucalypts (e.g., *Eucalyptus camaldulensis r* = 0.48, Butcher et al. 2009). Such a pattern in neutral genetic markers is expected under the isolation-by-distance model (Wright 1943) and indicates that drift is a key driver of the patterns of genetic variation. As in most eucalypts (Byrne 2008), gene flow in *E. globulus* is likely to be more a function of pollen than seed dispersal (Jones et al. 2007). Nevertheless, most pollen appears to be deposited within 100 m of the source (Mimura et al. 2009). Such distance is many orders of magnitude smaller than the geographic distance over which *E. globulus* is continuously distributed, providing the conditions for isolation-by-distance to occur. At a finer geographic scale, there is evidence to suggest that drift has been accentuated in small, geographically or ecologically isolated populations, resulting in strong divergence from the rest of the *E. globulus* gene pool.

Genetic drift and limited gene flow are key processes underlying the central-peripheral hypothesis, which predicts that populations at the margins of a species' range should exhibit lower genetic diversity and greater genetic differentiation than in the central part of the range (reviewed by Eckert et al. 2008). This hypothesis appears to hold at least in *bicostata*, with highest diversity in the continuous, central part of the range and lower diversity at the edge and in disjunct regions in the north and west of the range. For the species complex as a whole, though, high levels of genetic diversity were detected in the southeastern rather than the central part of the range. High genetic diversity is typically used as evidence for a center of origin (Vavilov 1992), suggesting that the southeastern region is the origin of the species complex. High genetic diversity in northern populations of the *Acacia saligna* species complex in Western Australia was evidence for a center of diversity or refugium (Millar et al. 2011), and in *E. camaldulensis*, 40% of the variation in allelic richness could be explained by latitude, with lower diversity in the south (Butcher et al. 2009), supporting the suggestion that the southern populations originated from the northern red gums (Hope 1994). A strong relationship between latitude and mean number of alleles per allozyme locus was also shown in populations of *Eucalyptus obtusiflora* (*r* = 0.91, Kennington and James 1998), a widespread species with a linear distribution pattern around 500-km long by 100-km wide. The non-linear/non-radial migration patterns and evolutionary history of *E. globulus* (Freeman et al. 2001) and the geographic barriers to dispersal that divide its distribution (the Murray Darling Depression, the highlands of the Great Dividing Range and Bass Strait, see Fig. 2) could account for the fact that the relationship between distance from South East Forests and heterozygosity/allelic richness was not strong. Two lines of evidence give further strength to the hypothesis that the South East Forests region is the origin of the species complex: (1) the Neighbour-Joining tree, in which the South East Forests populations cluster centrally; and (2) the capsule morphology in this region, which is assumed to be ancestral (Jordan et al. 1993), as seven-fruited umbels occur in other members of the subseries *Globulinae,* but single-fruited umbels, as in *globulus*, do not (Slee et al. 2006).

### Hybridization

There is good evidence that hybridization with species outside the *E. globulus* complex has affected the *E. globulus* gene pool, although nuclear DNA marker studies (McKinnon et al. 2010) indicate that this may be less extensive than was initially predicted from chloroplast markers (Jackson et al. 1999; McKinnon et al. 2001, 2004b). Similarly, this study provides evidence to suggest that hybridization and introgression among differentiated components of the *E. globulus* complex may have occurred in parts of the geographic range (e.g., 20-Strzelecki Ranges, see below), but most of the intergrade populations appear to be a result of primary differentiation as suggested by Kirkpatrick (1975). This is inferred from the absence of a peak in genetic diversity and the general lack of correlation between morphological and molecular affinities in intergrade zones. However, it is difficult to distinguish primary differentiation from ancient secondary contact (Endler 1977). It is sometimes achieved, using a combination of nuclear DNA markers and uniparentally inherited cytoplasmic DNA evidence (e.g., McKinnon et al. 2010). However, this has not been possible in *E. globulus* intergrade populations because of the confounding effects of potentially extensive chloroplast capture from species outside the *E. globulus* complex that can result in chloroplast DNA variation among species being correlated with geography rather than species boundaries (Steane et al. 1998; McKinnon et al. 1999, 2001, 2004a).

Although primary differentiation and ancient secondary intergradation may be difficult to distinguish, there is evidence to support the occurrence of recent secondary intergradations in *E. globulus*. A zone of recent secondary intergradation would be expected to display increased microsatellite diversity (due to gene flow from differentiated populations; Rieseberg and Wendel 1993) and a correlation between morphological and molecular affinities, as was the case in the *Q. crassifolia*–*Q. crassipes* hybrid zone in Mexico (Tovar-Sanchez and Oyama 2004). Of all the intergrade regions that were analyzed, only the 20-Strzelecki Ranges exhibited a significant correlation between morphological and molecular affinities, but showed no increase in genetic diversity compared with neighboring regions. A previous study of this region did not detect any microsatellite differentiation between individuals with *globulus* and *bicostata* morphological affinity and concluded that there was no evidence of recent secondary contact and introgression (Jones et al. 2002). This study, using a more sensitive analysis and greater sampling of the subspecies cores, provides evidence for recent gene flow from the adjacent coastal *globulus* into the Strzelecki Ranges population.

There is also the possibility of secondary gene flow across the eastern Bass Strait, between the Tasmanian *globulus* and the *pseudoglobulus* genetic groups. Tasmania and the mainland would have been linked by a land bridge formed following the lowering of sea levels during the Pleistocene glacial periods (McKinnon et al. 2004a). There are shared chloroplast DNA haplotypes (Freeman et al. 2001, 2007) and nuclear microsatellite (Steane et al. 2006; Yeoh et al. 2012; present study) affinities between western Victoria, western Tasmania, and King Island that support a western seed-dispersal link across this land bridge. However, although eastern mainland and Tasmanian choroplast DNA haplotypes occur on the Furneaux Group of islands, these haplotypes do not span the Bass Strait, thereby arguing for an eastern barrier to seed dispersal (Freeman et al. 2001, 2007). Nevertheless, pollen-mediated gene flow between eastern Victoria, Furneaux Group and northeast Tasmanian populations is suggested by the sharing of a rare haplotype of the nuclear *CCR* gene (McKinnon et al. 2005), as well as the occasional occurrence of mainland leaf chemotypes (Wallis et al. 2011) and the *pseudoglobulus* microsatellite group in the Furneaux Group and Northeastern Tasmania (*K* = 6 in Fig. 4). The far northern populations in the Furneaux group have morphological affinities to *pseudoglobulus*, and Jordan et al. (1993) have hypothesized that the Furneaux Group may be a zone of secondary contact between Tasmanian *globulus* and the *pseudoglobulus* genetic groups. Indeed, Wallis et al. (2011) hypothesized that on-going pollen-mediated gene flow between Tasmania and the mainland occurs along this eastern route, facilitated by the migratory swift parrot (*L. discolor*), a major pollinator of *globulus* (Hingston et al. 2004). Nevertheless, this study shows no evidence of increased nuclear microsatellite marker variability in the Furneaux Group expected from recent contact between well-differentiated gene pools.

### Natural selection

At various geographic locations across the range of the *E. globulus* complex, there is clear evidence for selection on capsule morphology characters, which are the key taxonomic traits. The lack of association between morphological and geographic distance is evidence for selection on capsule morphology traits, and while the association between genetic distance and morphological distance among regions was significant, it was small and there were a number of key correlation breakers where there was a discrepancy between morphology and neutral markers. For example, close morphological affinities but large genetic distances between 1-Mt. Bryan and the other *bicostata* regions are evidence not only for genetic drift but also for selection maintaining the *bicostata* capsule morphology despite migration over large geographic distances. Conversely, the 21-South Gippsland region of core *globulus* morphology had molecular affinities to geographically close, but morphologically distant regions, such as 20-Strzelecki Ranges, 15-Buchan, and the *pseudoglobulus* regions. The presence of these correlation breakers suggests that selection for large capsules over a steep environmental gradient has occurred in the Gippsland area (Jones et al. 2002). There is a positive correlation between seed size and capsule size at the population level within *E. globulus* (McGowen et al. 2004). Seed size influences seedling growth and bigger/heavier seed has been shown to result in faster growth of *E. globulus* during seedling establishment (Lopez et al. 2003). Selection for large capsules could, thus, have occurred in dry coastal areas as, for example, a mechanism to protect seeds during intense fire (Murray and Gill 2001) or to produce larger seed required for establishment in drought-prone areas (Hallett et al. 2011). Selection could, thus, explain discrepancies between morphology and microsatellite affinities, and it is noteworthy that several key discrepancies at the population level involve deviation of the molecular lineage in the direction of larger capsules and fewer capsules per inflorescence. This trend is evident, for example, in the *maidenii* molecular lineage at 7-Jenolan and in the *pseudoglobulus* molecular lineages at 21-South Gippsland and 2-Mt. Cole.

With its broad native range, the *E. globulus* complex spans diverse climatic zones in Australia ranging from summer (*maidenii*) to winter (*bicostata*, *globulus*) rainfall zones (Jordan et al. 1993). *Eucalyptus globulus* provenance trials established within and outside the native range of the complex in Australia have shown adaptive divergence at various geographic scales, from subspecies and regions (races) to local population level, indicating that natural selection has also played a significant role in shaping variation patterns in the *E. globulus* complex. At the subspecies level, core *bicostata* and *maidenii* are poorly adapted to the cool temperate climate of Tasmania (Volker and Orme 1988; Kube et al. 1995), whereas *globulus* is clearly more susceptible to leaf disease than *maidenii* (Carnegie et al. 1994; Hood et al. 2002), a factor contributing to the mal-adaptation of *globulus* to summer rainfall zones (Carnegie et al. 1994).

Of the four subspecies, *globulus* and its intergrades are the most intensively studied (because of their commercial importance; Dutkowski and Potts 1999; Stackpole et al. 2011) and there is plentiful evidence of broad-scale differential adaptation among races in many quantitative traits. By comparing differentiation of putatively neutral molecular (*F*_ST_) and quantitative (*Q*_ST_) traits, Steane et al. (2006) identified a number of phenotypic traits that had been influenced by diversifying selection (*Q*_ST_ > *F*_ST_). Subsequent studies have revealed other traits where *Q*_ST_ > *F*_ST_ (Hamilton et al. 2011; Stackpole et al. 2011; Dutkowski and Potts 2012). Many of these traits show further evidence of adaptation, as their variation is associated with broad-scale (Stackpole et al. 2011; Dutkowski and Potts 2012) or local (Jordan et al. 2000; Foster et al. 2007; Dutkowski and Potts 2012) environmental gradients.

### Practical implications

The molecular perspective on the *E. globulus* species complex is consistent with the taxonomic and morphological treatment in many respects. For example, while there is some admixture in intergrade populations, the cores of three of the four recognized taxa (*globulus*, *bicostata,* and *maidenii*) are relatively well-defined genetic entities, consistent with their geographic separation. The key problem is the lack of morphological definition of a large fraction of the gene pool that includes *pseudoglobulus* and the populations south of the Great Dividing Range in Victoria that comprise morphological intergrades between the cores (Jordan et al. 1993). In this region, there is little relationship between morphological and molecular affinities; yet at the molecular level, populations in this region form a relatively well-defined group that is genetically and geographically intermediate between the other three genetic groups. The taxonomic treatment of the populations from this “cryptic genetic entity” has been historically variable, which has caused confusion for conservation and exploitation of this gene pool. For example, different breeding programs have allocated the intergrade 20-Strzelecki region to different taxa (Jones et al. 2002). On the other hand, some populations classified morphologically as *globulus* in southern Gippsland belong to this cryptic genetic entity. From a practical perspective, the simplest higher level summary of the genetic diversity in this complex would be to expand the definition of *pseudoglobulus* to fully encompass populations in the cryptic genetic entity. The definition of taxa based on molecular data may be taxonomically challenging and difficult to apply. However, in this case, the genetic entity can be well defined geographically within Victoria, with the exception of a few peripheral populations (e.g. 2-Mt. Cole, 13-East Gippsland). Thus, geographic location would be the most pragmatic approach to classify germplasm in this region into taxonomic units for breeding and conservation purposes.

The study has provided a molecular framework for the management of the genetic resources of the *E. globulus* complex from several perspectives. First, the genetic entities defined at various levels with the STRUCTURE analysis can guide translocation of *E. globulus* germplasm within its native range for forestry, agro-forestry, and restoration purposes. The level of molecular differentiation between a local and a non-local seed source can provide an early predictor of the genetic impact of introgression between translocated and native populations (Potts et al. 2003). This is because the more the populations differ, the more likely new genes are to be introgressed into the native population. Assessing the long-term consequences of introgression from tree plantings is extremely difficult given the long generation times and such information is one component of assessing genetic risk associated with translocation (Laikre et al. 2010; Byrne and Stone 2011; Byrne et al. 2011). Second, *globulus*, together with some Victorian intergrade populations, which are here classified with *pseudoglobulus*, forms the base populations of *E. globulus* breeding programs in many countries (Potts et al. 2004). As there are close molecular relationships among most Victorian populations, and breeding programs already include populations from the cryptic genetic entity identified here (Jones et al. 2006), a broader range of Victorian populations could readily be infused into the current breeding programs. Finally, molecular relationships among populations can be used to improve quantitative genetic evaluation of germplasm in breeding programs, because usual practice assumes unrelated genetic groups of founder individuals. In the Australian *E. globulus* breeding program, the subrace classification of Dutkowski and Potts (1999) is used to define genetic groups in these evaluations (McRae et al. 2004). However, molecular studies (Steane et al. 2006; Yeoh et al. 2012; this study) suggest that this grouping is conservative and, as discussed by Steane et al. (2006), some subraces are more closely related than others, which could be accounted for in quantitative genetic evaluations.

## Supporting Information

**Table S1.** Sample sizes (*n*) of *Eucalyptus globulus* localities in this study, after excluding individuals that were missing data from three or more loci.

**Table S2.** Overall genetic diversity parameters for the microsatellite loci used in the analysis of the *Eucalyptus globulus* species complex.

**Figure S1.** Calculation of Δ*K*, following Evanno et al. (2005).

**Data S1.** Description of the repeatability of scoring for the microsatellite loci used in the analysis of the *Eucalyptus globulus* species complex.

**Data S2.** Description of the overall genetic diversity parameters for the microsatellite loci used in the analysis of the *Eucalyptus globulus* species complex.
