# Supplementary material for: Multiple evolutionary processes drive the patterns of genetic differentiation in a forest tree species complex
Source: Ecol Evol. 2013 Jan 10;3(1):1–17. doi: 10.1002/ece3.421 (PMC3568837; doi:10.1002/ece3.421)
Supplement: Supplementary file 1 [file ece30003-0001-SD2.docx]

Table S1. Sample sizes (*n*) of *Eucalyptus globulus* localities in this study, after excluding individuals that were missing data from three or more loci. B = *bicostata*, g = *globulus* intergrade, G = *globulus*, M = *maidenii*, m = *maidenii* – *pseudoglobulus* intergrade, P = *pseudoglobulus*, b = *bicostata*-*pseudoglobulus* intergrade. The taxon codes of “core” localities, used as *a priori* groupings for comparison of morphological and molecular affinities of intergrade samples, and to calculate pairwise *F*_ST_ values among taxa, are underlined.

| Taxon | Region code / name | Locality code / name | Latitude | Longitude | Altitude | n |
| --- | --- | --- | --- | --- | --- | --- |
| B | 1 Mt Bryan | 1 Mt Bryan^1^ | 33.44 | 138.96 | 821 | 25 |
| B | 2 Mt Cole | 2 Mt Cole^1^ | 37.30 | 143.24 | 826 | 19 |
| B | 3 Hume | 3 Tallarook^1,2^ | 37.15 | 145.12 | 479 | 9 |
| B | 3 Hume | 4 Euroa^2^ | 36.82 | 145.69 | 600 | 12 |
| B | 3 Hume | 5 Eildon^2^ | 37.31 | 146.09 | 850 | 9 |
| B | 4 Omeo | 6 Beloka Rd^1^ | 36.89 | 147.82 | 953 | 7 |
| B | 4 Omeo | 7 Omeo^1^ | 37.12 | 147.56 | 763 | 8 |
| B | 4 Omeo | 8 Trapyard Gap^1^ | 36.94 | 147.43 | 1096 | 7 |
| B | 5 North-eastern Victoria | 9 Mt Granya^1^ | 36.13 | 147.27 | 867 | 8 |
| B | 5 North-eastern Victoria | 10 Shelley^1^ | 36.18 | 147.55 | 754 | 8 |
| B | 5 North-eastern Victoria | 11 Nariel^1^ | 36.48 | 147.80 | 1029 | 8 |
| B | 5 North-eastern Victoria | 12 Mt Unicorn^1^ | 36.34 | 147.97 | 843 | 8 |
| B | 6 Canberra | 13 Bungongo^1^ | 35.04 | 148.48 | 791 | 13 |
| B | 6 Canberra | 14 Burrinjuck^1^ | 34.97 | 148.63 | 505 | 7 |
| B | 7 Jenolan | 15 Jenolan^1^ | 33.82 | 150.03 | 771 | 22 |
| B | 8 Nullo Mountain | 16 Nullo Mountain^1^ | 32.74 | 150.22 | 1102 | 20 |
| M | 9 Araluen | 17 Araluen^1^ | 35.61 | 149.79 | 471 | 16 |
| M | 9 Araluen | 18 Currowan-Monga^1^ | 35.61 | 150.05 | 383 | 23 |
| M | 10 Wadbilliga | 19 Wadbilliga North^1^ | 36.13 | 149.63 | 353 | 14 |
| M | 10 Wadbilliga | 20 Belowra Rd^1^ | 36.16 | 149.84 | 492 | 10 |
| M | 10 Wadbilliga | 21 Wadbilliga South^1^ | 36.25 | 149.64 | 329 | 20 |
| M | 10 Wadbilliga | 22 Murrabrine^1^ | 36.36 | 149.74 | 428 | 20 |
| M | 10 Wadbilliga | 23 Kooraban NP^1^ | 36.34 | 149.88 | 247 | 6 |
| M | 10 Wadbilliga | 24 Mt Dromedary (NSW)^1^ | 36.29 | 150.04 | 220 | 13 |
| M | 10 Wadbilliga | 25 Mumbulla SF^1^ | 36.58 | 149.86 | 185 | 20 |
| M | 10 Wadbilliga | 26 Brown Mountain^1^ | 36.61 | 149.47 | 436 | 12 |
| M | 11 South-East Forests | 27 Tantawangalow^1^ | 36.80 | 149.58 | 299 | 8 |
| M | 11 South-East Forests | 28 Big Jack - Rocky Hall^1^ | 36.89 | 149.46 | 357 | 15 |
| M | 11 South-East Forests | 29 Yurammie^1^ | 36.90 | 149.72 | 480 | 16 |
| M | 11 South-East Forests | 30 Mt Imlay^1^ | 37.12 | 149.69 | 316 | 8 |
| M | 11 South-East Forests | 31 North Cann Valley Highway^1^ | 37.27 | 149.23 | 298 | 7 |
| M | 11 South-East Forests | 32 Wroxham^1^ | 37.34 | 149.47 | 297 | 8 |
| M | 11 South-East Forests | 33 Maramingo Creek^1^ | 37.42 | 149.62 | 173 | 10 |
| m | 12 Alfred-Nadgee | 34 Nadgee^1^ | 37.44 | 149.96 | 14 | 18 |
| m | 12 Alfred-Nadgee | 35 Alfred NP^1^ | 37.57 | 149.34 | 331 | 9 |
| m | 12 Alfred-Nadgee | 36 Mid Cann Valley Highway^1^ | 37.43 | 149.20 | 151 | 6 |
| m | 12 Alfred-Nadgee | 37 South Cann Valley Highway^1^ | 37.50 | 149.18 | 132 | 12 |
| P | 13 East Gippsland | 38 Mt Cann^1^ | 37.65 | 148.96 | 311 | 10 |
| P | 13 East Gippsland | 39 Wiebens Hill^1,2^ | 37.61 | 148.78 | 282 | 16 |
| P | 14 Lakes Entrance | 40 Ostlers Rd^1,2^ | 37.80 | 148.04 | 43 | 9 |
| P | 14 Lakes Entrance | 41 Lake Tyers^1,2^ | 37.86 | 148.04 | 25 | 14 |
| P | 14 Lakes Entrance | 42 Lakes Entrance^1,2^ | 37.88 | 147.97 | 41 | 11 |
| P | 14 Lakes Entrance | 43 Metung-Lakes Entrance^1,2^ | 37.88 | 147.89 | 43 | 6 |
| Taxon | Region code / name | Locality code / name | Latitude | Longitude | Altitude | n |
| P | 14 Lakes Entrance | 44 Metung^1,2^ | 37.88 | 147.86 | 24 | 8 |
| b | 15 Buchan | 45 Stoney Creek^1,2^ | 37.57 | 148.26 | 241 | 10 |
| b | 15 Buchan | 46 Cutts Creek Rd^1^ | 37.47 | 148.01 | 438 | 8 |
| b | 15 Buchan | 47 Buchan^1,2^ | 37.45 | 148.19 | 339 | 11 |
| b | 15 Buchan | 48 Gelantipy^1^ | 37.27 | 148.25 | 746 | 8 |
| b | 16 Mitchell River | 49 Cobbannah^1^ | 37.69 | 147.29 | 400 | 10 |
| b | 16 Mitchell River | 50 Peel Gap^1^ | 37.59 | 147.18 | 514 | 10 |
| b | 17 Lerderderg | 51 Lerderderg^1^ | 37.61 | 144.42 | 153 | 20 |
| g (Vic) | 18 Eastern Otways | 52 Lorne PO^3^ | 38.53 | 143.97 | 61 | 14 |
| g (Vic) | 18 Eastern Otways | 53 Jamieson Creek^3^ | 38.60 | 143.91 | 173 | 8 |
| g (Vic) | 18 Eastern Otways | 54 Cape Patton^3^ | 38.67 | 143.85 | 88 | 7 |
| g (Vic) | 19 Western Otways | 55 Parker Spur^3^ | 38.81 | 143.56 | 171 | 8 |
| g (Vic) | 19 Western Otways | 56 Cannan Spur^3^ | 38.76 | 143.54 | 204 | 8 |
| g (Vic) | 19 Western Otways | 57 Otways State Forest^3^ | 38.78 | 143.42 | 68 | 8 |
| g (Vic) | 19 Western Otways | 58 S.W. Lavers Hill^3^ | 38.74 | 143.25 | 134 | 8 |
| g (Vic) | 20 Strzelecki Ranges | 59 Bowden Road^2,7^ | 38.42 | 146.68 | 422 | 1 |
| g (Vic) | 20 Strzelecki Ranges | 60 Jeeralang^2,7^ | 38.41 | 146.53 | 443 | 4 |
| g (Vic) | 20 Strzelecki Ranges | 61 Jeeralang North^2^ | 38.36 | 146.49 | 140 | 19 |
| G (Vic) | 21 South Gippsland | 62 Alberton West^2,7^ | 38.62 | 146.53 | 62 | 4 |
| G (Vic) | 21 South Gippsland | 63 Hedley^2^ | 38.64 | 146.49 | 27 | 8 |
| G (Vic) | 21 South Gippsland | 64 Welshpool^2,7^ | 38.67 | 146.45 | 14 | 2 |
| G (Vic) | 21 South Gippsland | 65 Toora^2,7^ | 38.67 | 146.28 | 20 | 4 |
| G (Vic) | 21 South Gippsland | 66 Port Franklin^2,7^ | 38.67 | 146.27 | 11 | 4 |
| G (Vic) | 21 South Gippsland | 67 Fish Creek^2^ | 38.72 | 146.10 | 93 | 6 |
| G (Vic) | 22 Phillip Island | 68 Phillip Island^2,6^ | 38.48 | 145.26 | 32 | 24 |
| G (Vic) | 23 Tidal River | 69 Tidal River^3^ | 39.02 | 146.34 | 20 | 31 |
| G (Vic) | 24 Wilson's Promontory Lighthouse | 70 Wilson's Promontory Lighthouse^4^ | 39.12 | 146.42 | 139 | 31 |
| g (Tas) | 25 King Island | 71 King Island North^3^ | 39.65 | 144.04 | 51 | 6 |
| g (Tas) | 25 King Island | 72 Central King Island North^3^ | 39.76 | 143.98 | 39 | 5 |
| g (Tas) | 25 King Island | 73 King Island Central West^3^ | 39.93 | 143.96 | 91 | 13 |
| g (Tas) | 25 King Island | 74 Central King Island East^3^ | 39.94 | 144.07 | 98 | 8 |
| g (Tas) | 25 King Island | 75 King Island South West^3,7^ | 40.05 | 143.94 | 92 | 1 |
| g (Tas) | 25 King Island | 76 South King Island East ^3^ | 40.03 | 144.06 | 94 | 7 |
| g (Tas) | 26 Western Tasmania | 77 Little Henty River^3^ | 41.94 | 145.20 | 58 | 8 |
| g (Tas) | 26 Western Tasmania | 78 Badgers Creek^3^ | 42.00 | 145.28 | 115 | 8 |
| g (Tas) | 26 Western Tasmania | 79 Macquarie Harbour^3^ | 42.34 | 145.33 | 3 | 16 |
| g (Tas) | 27 Port Davey | 80 Port Davey^3^ | 43.30 | 145.92 | 19 | 35 |
| G (Tas) | 28 Recherche Bay | 81 Recherche Bay^3,6^ | 43.53 | 146.90 | 23 | 27 |
| G (Tas) | 29 Southern Tasmania | 82 South Bruny Island^3^ | 43.35 | 147.32 | 5 | 8 |
| G (Tas) | 29 Southern Tasmania | 83 Dover^3^ | 43.31 | 147.07 | 67 | 7 |
| G (Tas) | 29 Southern Tasmania | 84 South Geeveston^3^ | 43.19 | 146.90 | 100 | 10 |
| G (Tas) | 30 South-eastern Tasmania | 85 Tinderbox^5^ | 43.03 | 147.32 | 300 | 9 |
| G (Tas) | 30 South-eastern Tasmania | 86 Hobart South^3,7^ | 42.91 | 147.29 | 296 | 1 |
| G (Tas) | 30 South-eastern Tasmania | 87 South Maria Island^4^ | 42.72 | 148.07 | 82 | 28 |
| G (Tas) | 30 South-eastern Tasmania | 88 North Maria Island^4^ | 42.59 | 148.07 | 62 | 8 |
| G (Tas) | 30 South-eastern Tasmania | 89 Triabunna^3^ | 42.46 | 147.90 | 265 | 8 |
| G (Tas) | 30 South-eastern Tasmania | 90 Platform Peak^3^ | 42.68 | 147.03 | 539 | 17 |
| G (Tas) | 31 Dromedary | 91 Mt Dromedary^3^ | 42.72 | 147.13 | 389 | 18 |
| G (Tas) | 32 North-eastern Tasmania | 92 Jericho^3^ | 42.43 | 147.26 | 413 | 6 |
| G (Tas) | 32 North-eastern Tasmania | 93 Pepper Hill^3^ | 41.64 | 147.85 | 602 | 8 |
| G (Tas) | 32 North-eastern Tasmania | 94 Mayfield^3^ | 42.21 | 148.03 | 50 | 6 |
| G (Tas) | 32 North-eastern Tasmania | 95 Mayfield North^3^ | 42.02 | 147.97 | 296 | 8 |
| G (Tas) | 32 North-eastern Tasmania | 96 Cape Tourville Dwarf^4^ | 42.12 | 148.34 | 122 | 20 |
| G (Tas) | 32 North-eastern Tasmania | 97 Cape Tourville Tall^4^ | 42.12 | 148.34 | 99 | 38 |
| G (Tas) | 32 North-eastern Tasmania | 98 St Helens^3^ | 41.27 | 148.30 | 139 | 9 |
| G (Tas) | 33 Furneaux | 99 North Cape Barren Island^3^ | 40.34 | 148.26 | 12 | 27 |
| G (Tas) | 33 Furneaux | 100 South Flinders Island^3,7^ | 40.21 | 148.24 | 15 | 2 |
| Taxon | Region code / name | Locality code / name | Latitude | Longitude | Altitude | n |
| G (Tas) | 33 Furneaux | 101 Central Flinders Island^3^ | 40.03 | 147.99 | 35 | 6 |
| G (Tas) | 33 Furneaux | 102 Central North Flinders Island^3^ | 39.95 | 147.99 | 92 | 9 |
| G (Tas) | 33 Furneaux | 103 North Flinders Island^3,7^ | 39.78 | 147.90 | 31 | 2 |

Samples obtained from ^1^This study; ^2^Jones et al. 2002; ^3^Steane et al. 2006; ^4^Foster et al. 2007; ^5^Jones et al. 2007; ^6^ Steane et al in prep. ^7^Due to small sample size, these localities were excluded from analyses at the locality level, but were included in the STRUCTURE and regional level analyses.
