# Supplementary material for: Multiple evolutionary processes drive the patterns of genetic differentiation in a forest tree species complex
Source: Ecol Evol. 2013 Jan 10;3(1):1–17. doi: 10.1002/ece3.421 (PMC3568837; doi:10.1002/ece3.421)
Supplement: Supplementary file 2 [file ece30003-0001-SD3.pdf]

Table S2. Overall genetic diversity parameters for the microsatellite loci used in the analysis of the *Eucalyptus globulus* species complex. *n* = number of individual trees. The number of individual trees varies among loci because clear amplification products were not obtained for all individuals. *A* = observed number of alleles per locus, H_e = expected heterozygosity, H_o = observed heterozygosity, *F* = Wright's Fixation Index. Null = frequency of null alleles, averaged over regions. Inbreeding coefficients (F_{IS}, F_{IT}, F_{ST}) were calculated for each locus at the regional level, based on the 33 regions of the *E. globulus* complex (see Table S1). The 95% confidence intervals on the mean inbreeding coefficients, derived from 1000 bootstraps, are given in parentheses.

Locus	*n*	Size range (bp)	*A*	H_e	H_o	*F*	Null	F_{IS}	F_{IT}	F_{ST}
EMBRA11	1151	88-164	35	0.92	0.78	0.15	0.03	0.08	0.15	0.08
EMBRA19	1152	141-201	21	0.81	0.49	0.40	0.12	0.32	0.40	0.13
EMBRA30	1161	81-161	34	0.92	0.79	0.14	0.01	0.05	0.14	0.10
EMCRC2	1138	155-213	26	0.89	0.74	0.17	0.03	0.08	0.17	0.10
EMCRC5	1161	143-351	68	0.88	0.63	0.29	0.08	0.21	0.29	0.11
EMCRC6	1160	148-204	26	0.90	0.70	0.22	0.04	0.11	0.22	0.13
EMCRC7	1138	248-328	29	0.90	0.75	0.16	0.03	0.07	0.17	0.10
EMCRC10	1135	301-355	22	0.87	0.48	0.45	0.17	0.38	0.45	0.10
EMCRC11	1171	217-273	27	0.90	0.80	0.11	0.01	0.03	0.11	0.08
Mean	1152		32	0.89	0.69	0.23	0.06	0.14 (0.08-0.23)	0.23 (0.16-0.31)	0.10 (0.09-0.11)
