# Supplementary figures and images for: Multiple evolutionary processes drive the patterns of genetic differentiation in a forest tree species complex
Source: Ecol Evol. 2013 Jan 10;3(1):1–17. doi: 10.1002/ece3.421 (PMC3568837; doi:10.1002/ece3.421)

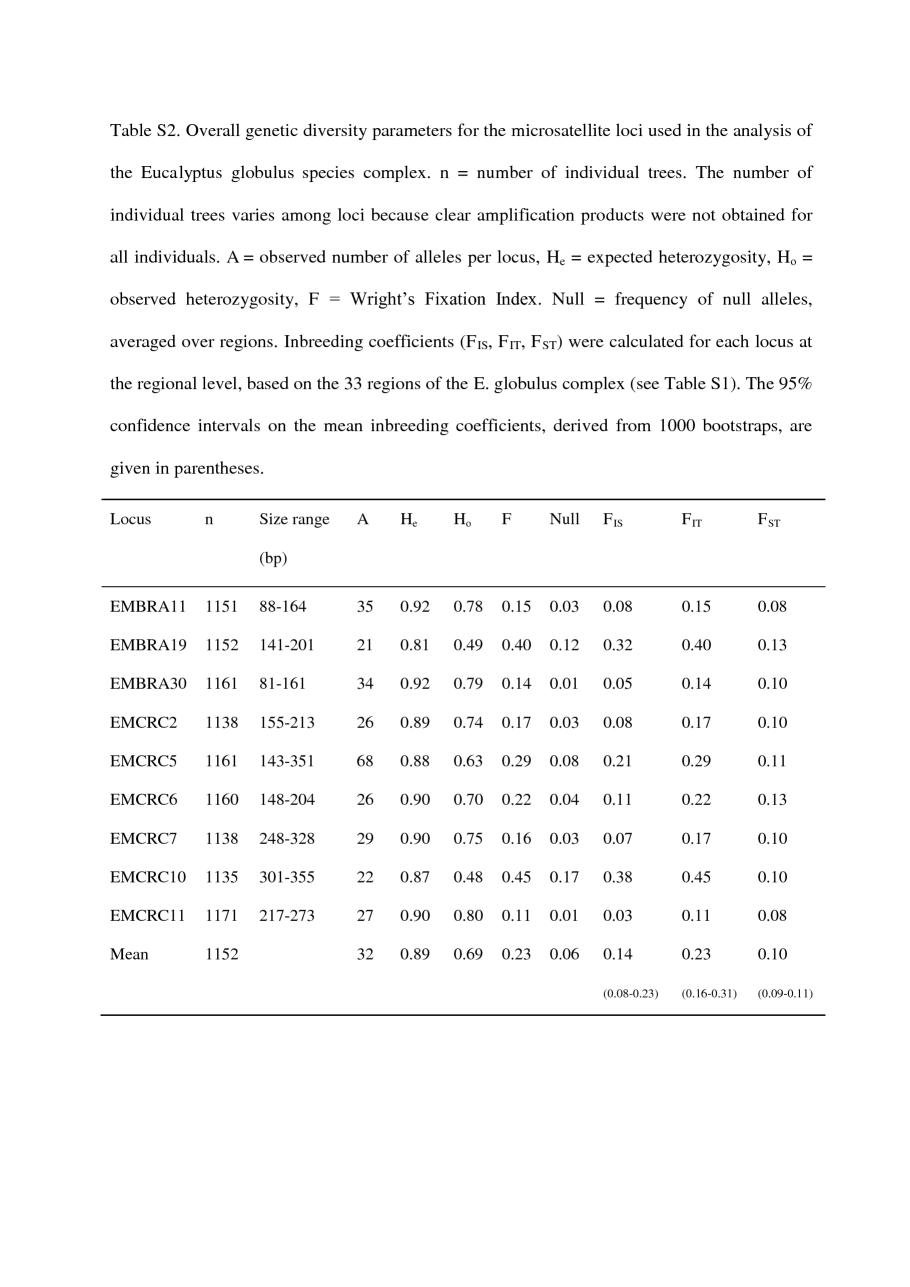

Supplement: Supplementary file 3 [file ece30003-0001-SD6.png]

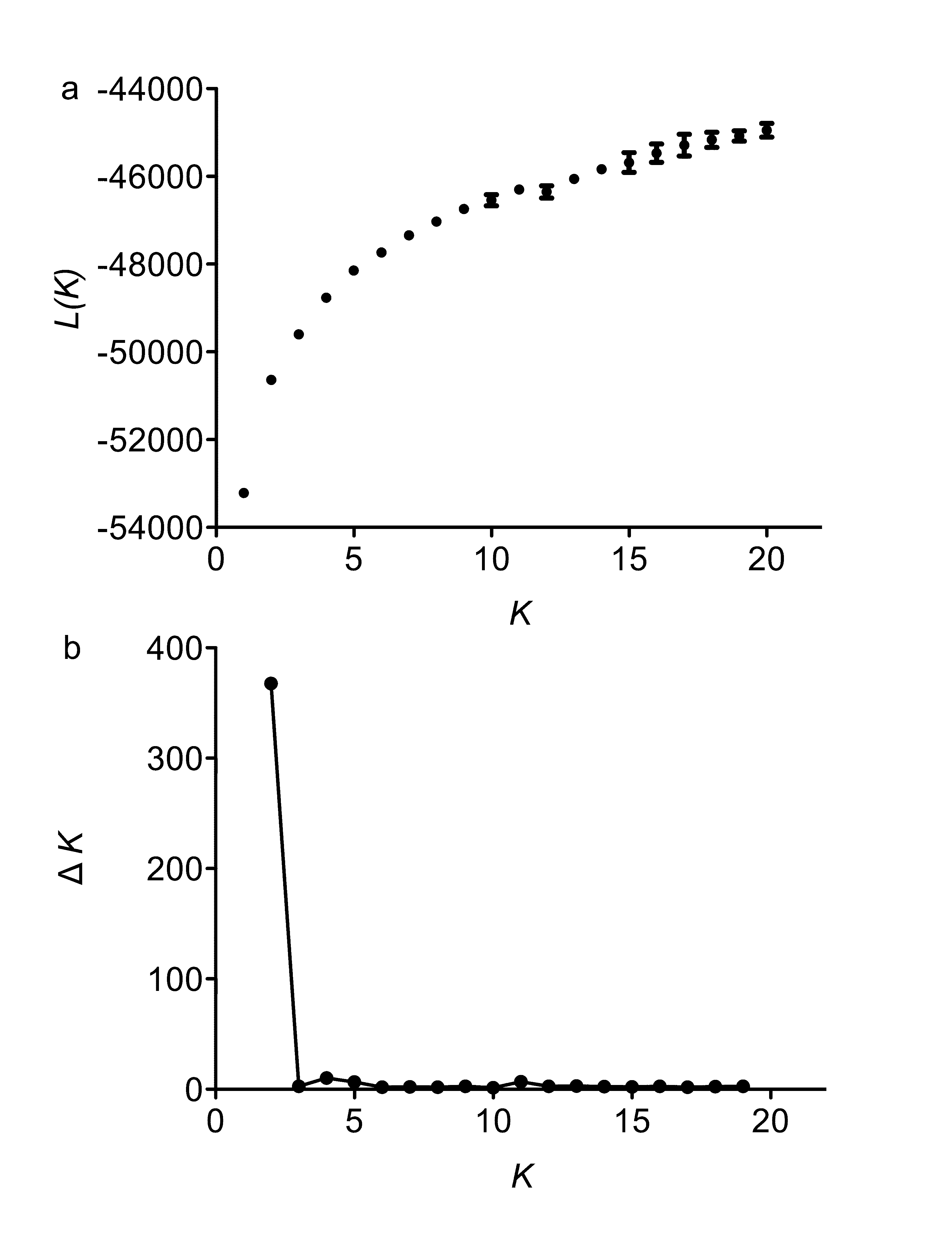

Supplement: Supplementary file 5 [file ece30003-0001-SD1.jpg]

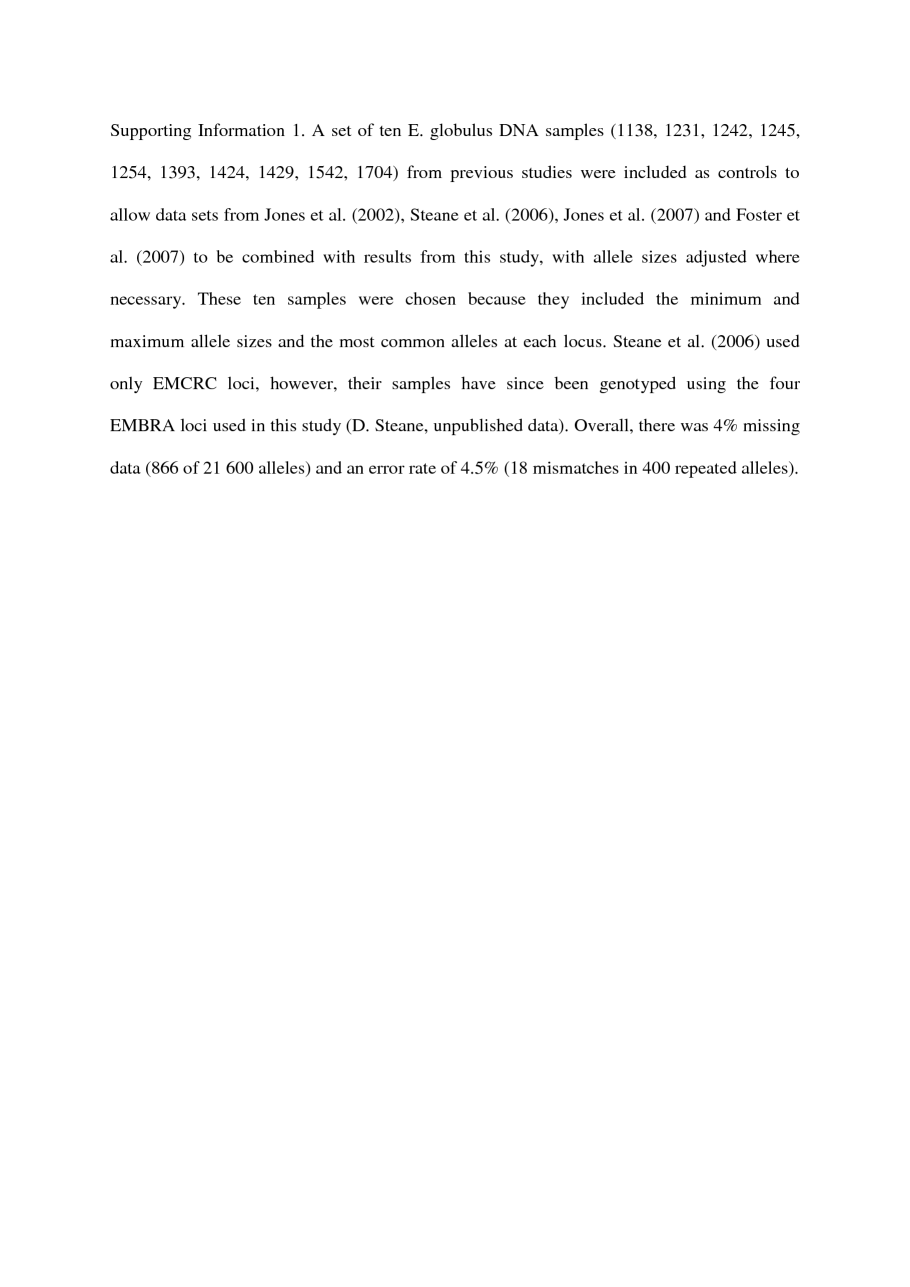

Supplement: Supplementary file 7 [file ece30003-0001-SD7.png]

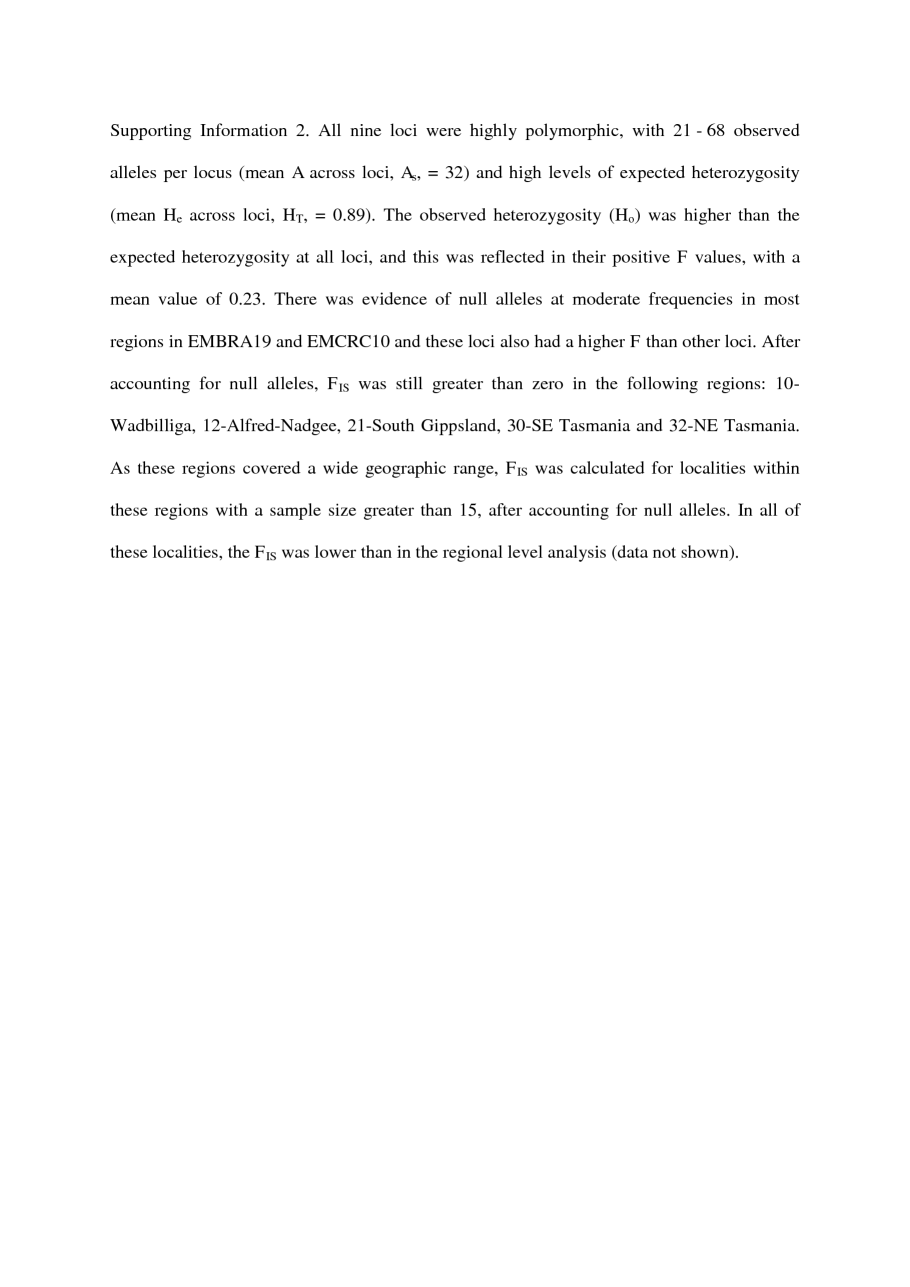

Supplement: Supplementary file 10 [file ece30003-0001-SD8.png]
