# Supplementary material for: Multiple evolutionary processes drive the patterns of genetic differentiation in a forest tree species complex
Source: Ecol Evol. 2013 Jan 10;3(1):1–17. doi: 10.1002/ece3.421 (PMC3568837; doi:10.1002/ece3.421)
Supplement: Supplementary file 6 [file ece30003-0001-SD4.pdf]

Supporting Information 1. A set of ten *E. globulus* DNA samples (1138, 1231, 1242, 1245, 1254, 1393, 1424, 1429, 1542, 1704) from previous studies were included as controls to allow data sets from Jones et al. (2002), Steane et al. (2006), Jones et al. (2007) and Foster et al. (2007) to be combined with results from this study, with allele sizes adjusted where necessary. These ten samples were chosen because they included the minimum and maximum allele sizes and the most common alleles at each locus. Steane et al. (2006) used only EMCRC loci, however, their samples have since been genotyped using the four EMBRA loci used in this study (D. Steane, unpublished data). Overall, there was 4% missing data (866 of 21 600 alleles) and an error rate of 4.5% (18 mismatches in 400 repeated alleles).
