# Supplementary material for: Multiple evolutionary processes drive the patterns of genetic differentiation in a forest tree species complex
Source: Ecol Evol. 2013 Jan 10;3(1):1–17. doi: 10.1002/ece3.421 (PMC3568837; doi:10.1002/ece3.421)
Supplement: Supplementary file 9 [file ece30003-0001-SD5.pdf]

Supporting Information 2. All nine loci were highly polymorphic, with 21 - 68 observed alleles per locus (mean A across loci, A_s, = 32) and high levels of expected heterozygosity (mean H_e across loci, H_T, = 0.89). The observed heterozygosity (H_o) was higher than the expected heterozygosity at all loci, and this was reflected in their positive F values, with a mean value of 0.23. There was evidence of null alleles at moderate frequencies in most regions in EMBRA19 and EMCRC10 and these loci also had a higher F than other loci. After accounting for null alleles, F_{IS} was still greater than zero in the following regions: 10-Wadbilliga, 12-Alfred-Nadgee, 21-South Gippsland, 30-SE Tasmania and 32-NE Tasmania. As these regions covered a wide geographic range, F_{IS} was calculated for localities within these regions with a sample size greater than 15, after accounting for null alleles. In all of these localities, the F_{IS} was lower than in the regional level analysis (data not shown).
